# An Information Theory Approach for the Analysis of Individual and Combined Evaluation Parameters of Multiple Age-Related Diseases

**DOI:** 10.3390/e21060572

**Published:** 2019-06-05

**Authors:** David Blokh, Ilia Stambler, Emilia Lubart, Eliyahu H. Mizrahi

**Affiliations:** 1 C.D. Technologies Ltd., Beer Sheba 8445914, Israel; 2 Department of Science, Technology and Society, Bar Ilan University, Ramat Gan 5290002, Israel; 3 The Geriatric Medical Center "Shmuel Harofe”, Beer Yaakov, Affiliated to Sackler School of Medicine, Tel-Aviv University, Tel-Aviv 6997801, Israel

**Keywords:** multimorbidity, aging, aging-related diseases, normalized mutual information, information theory

## Abstract

In view of the frequent presence of several aging-related diseases in geriatric patients, there is a need to develop analytical methodologies that would be able to perform diagnostic evaluation of several diseases at once by individual or combined evaluation parameters and select the most informative parameters or parameter combinations. So far there have been no established formal methods to enable such capabilities. We develop a new formal method for the evaluation of multiple age-related diseases by calculating the informative values (normalized mutual information) of particular parameters or parameter combinations on particular diseases, and then combine the ranks of informative values to provide an overall estimation (or correlation) on several diseases at once. Using this methodology, we evaluate a geriatric cohort, with several common age-related diseases, including cognitive and physical impairments (dementia, chronic obstructive pulmonary disease—COPD and ischemic heart disease), utilizing a set of evaluation parameters (such as demographic data and blood biomarkers) routinely available in geriatric clinical practice. This method permitted us to establish the most informative parameters and parameter combinations for several diseases at once. Combinations of evaluation parameters were shown to be more informative than individual parameters. This method, with additional clinical data, may help establish the most informative parameters and parameter combinations for the diagnostic evaluation of multiple age-related diseases and enhance specific assessment for older multi-morbid patients and treatments against old-age multimorbidity.

## 1. Introduction

Due to the global aging, the incidence of multiple aging-related diseases grows correspondingly [1]. Often, in geriatric patients, multiple aging-related diseases are present at once (a condition sometimes termed “multimorbidity”), which could be affected by multiple risk factors. There is thus a clear need to develop analytical methodologies that would be able to perform diagnostic evaluation of several diseases at once by individual or combined evaluation parameters and select the most informative individual or combined evaluation parameters (or risk factors). So far there have been no established formal methods to enable such capabilities, or even a consensus definition of “multimorbidity” [2]. Moreover, there have been no established formal methods to evaluate simultaneous changes in multiple age-related diseases as a result of single or combined therapeutic interventions. Such methodologies may be particularly valuable for attempting to create preventive treatments for multiple age-related diseases via intervention into their common underlying aging processes [3,4,5] and providing the formal evidence criteria for their effectiveness. Such measures of multiple diseases could also help developing a more holistic approach to treatment, careful not to treat one disease at the cost of aggravating another [6,7]. That is to say, such methodologies are needed to establish polygenic etiologies and multifactorial treatments for composite disease states [8]. Thus the formal capability to evaluate multiple age-related diseases (multimorbidity) from multiple determinants and risk factors, as well as changes in multiple diseases due to multiple therapeutic interventions, could be highly valuable both for diagnosis and therapy of older patients. Here we outline one of the possible directions toward developing such a methodological capability, using a convenient measure from information theory—the normalized mutual information (or uncertainty coefficient).

Generally, information-theoretical measures (such as normalized mutual information, as used here) could be instrumental for the evaluation of multiple age-related diseases from multiple biomarkers and other diagnostic parameters. Such an evaluation is enabled due to the capability of information-theoretical measures to establish the exact mutual influence (information content) between any set of parameters (e.g., a set of diagnostic biomarkers) with any set of parameters (e.g., diseases or conditions) as well as to establish their cumulative or synergistic effects [9]. Specifically, in the evaluation of multimorbidity, the following critical methodological problems emerge: First of all, there is a need to compare the influence of potential pathogenic factors on a group of diseases, and even more importantly to evaluate the combined influence of a group of potential pathogenic factors on a group of diseases, insofar as old-age multimorbidity is commonly due to multiple etiologic factors. Moreover, those etiologic factors can be discrete as well as continuous parameters. Furthermore, the relations between etiologic factors and diseases in biological systems are as a rule non-linear. Arguably, the mathematically grounded methodology that can enable the solution of such problems in the evaluation of multimorbidity is the information-theoretical methodology [9]. 

Yet, there may be several approaches to using information-theoretical measures (such as normalized mutual information) for the estimation of multiple age-related diseases or “multimorbidity.” In our earlier study, we developed a method to evaluate multimorbidity as a composite variable, composed of several age-related diseases—creating a kind of a new disease entity termed “multimorbidity” that assumes various states according to the presence or absence of particular diseases [10]. In the present study, we propose a different methodological approach, which evaluates the informative values of particular parameters or parameter combinations on particular diseases separately, and then combines the ranks of informative values to provide an overall estimation on several diseases at once. In other words, we first calculate the strength of correlation between the multiple parameters and single diseases, and thereafter with a group of diseases, using the information-theoretical measure of normalized mutual information. Informally put, we calculate, based on the values of individual or multiple evaluation parameters, the measure of precision for determining the simultaneous presence or absence of several diseases at once (or multimorbidity). This new methodological approach was established here on a geriatric cohort, suffering form several common age-related diseases (dementia, chronic obstructive pulmonary disease and ischemic heart disease), utilizing a set of evaluation parameters routinely available in geriatric clinical practice (see the “Materials and Methods” section). Yet, future research, involving additional samples, will determine which of the various possible methodological approaches to evaluate multiple age-related diseases (or old-age multimorbidity) is better suitable to serve clinical needs.

## 2. Materials and Methods

### 2.1. Mathematical Analysis

The general problem of this study could be stated as follows. If given multiple diseases and multiple biomarkers or other factors (evaluation parameters), we need to find, in the entire set of evaluation parameters, such parameters or combinations of parameters that contain the “most information” about the entire set of diseases compared to other parameters or combinations of parameters. An equivalent statement of the problem is that, out of the group of all the available evaluation parameters, we need to select such parameters or groups of parameters that can serve as the best indicators/markers (the most informative evaluation parameters) for multiple diseases simultaneously, according to the strength of correlation between individual parameters or groups of several parameters at the same time and a group of several diseases at the same time.

This problem is particularly pressing for elderly patients who, as a rule, have multiple diseases. The information-theoretical measure of normalized mutual information (or “uncertainty coefficient”) was selected as the measure of correlation between evaluation parameters (either individual or combined) with either individual or multiple diseases [9,10,11]. The methodological approach developed in Blokh et al. [11] was presented in the monograph [12]. The normalized mutual information is the precise quantitative measure of the amount of information that evaluation parameters (either individual or combined) have about a particular disease or multiple diseases.

We calculate the normalized mutual information in the following way. Let X be a discrete random variable with a distribution function as follows:

X   x_1_   x_2_  ....... x*_n_*;

Q   p_1_   p_2_  ....... p*_n_*.

X may be an evaluation parameter (a risk factor, a biomarker or a demographic characteristic), *n* may be the number of categories for this evaluation parameter (a risk factor, a biomarker or a demographic characteristic) and p_i_ indicates the frequency for the category x_i_. The entropy of the random variable X is determined by the formula:

$$H(X)=-\sum_{i=1}^{n}p_{i}\log p_{i}.$$

We assume X and Y to be discrete random variables (evaluation parameters, risk factors, biomarkers or other diagnostics). The algorithms used to calculate normalized mutual information between individual or combined evaluation variables have been presented earlier [9,13,14]. Essentially, for the evaluation variables X and Y, the value of normalized mutual information C is calculated by the formula:

$$C(X,Y)=\frac{I(X;Y)}{H(Y)}=\frac{H(X)+H(Y)-H(XY)}{H(Y)}.$$

Here the values H(X), H(Y) and H(XY) respectively indicate the entropies of random variables X, Y and X × Y.

The normalized mutual information has the following properties:

1. 0 ≤ C(X,Y) ≤ 1.
2. C(XY) = 0 if and only if the random variables X and Y are independent (no correlation or influence between the variables).
3. C(X,Y) = 1 if and only if there is a functional relation (correlation or influence) between X and Y.
4. C(X_1_,Y) ≤ C(X_1_X_2_,Y) and C(X_2_,Y) ≤ C(X_1_X_2_,Y). That is to say, the combined influence of two random variables X_1_ and X_2_ on a random variable Y is greater or equal to the influence of any of the random variables X_1_ or X_2_ on Y.

Note that the above four properties represent the established theoretical requirements necessary to be able to evaluate the correlation between random variables X and Y [15,16,17]. Presently, the only measure that satisfies all four requirements is the normalized mutual information (uncertainty coefficient) [15,16,17]. Hence the normalized mutual information was chosen here as the theoretically grounded, and thus preferable measure to evaluate correlations between random variables.

The normalized mutual information nearer to zero shows a weaker correlation between parameters, in other words, little information that one parameter contains about another. On the other hand, the normalized mutual information nearer to unity shows a stronger correlation between parameters, or a large amount of information that one parameter contains about another.

In order to determine the mutual correlation between a combined evaluation parameter (a combined biomarker or risk factor or demographic or some other diagnostic) and an individual disease, we initially calculated the general (combined) correlation of all the evaluation parameters composing the combined evaluation parameter with that individual disease. For a combined evaluation parameter comprised of two individual parameters, this is accomplished by the following algorithm: Let the combined diagnostic parameter X be composed of two discrete parameters X_1_ and X_2_ where the parameter X_1_ assumes two values: 0 and 1, and the parameter X_2_ assumes three values: 0, 1 and 2. Then the mutual relation of the combined parameter X with the individual disease is determined as the relation of an “individual evaluation parameter” assuming six values according to the values of the individual evaluation parameters X_1_ and X_2_: (0,0)—0, (0,1)—1, (0,2)—2, (1,0)—3, (1,1)—4 and (1,2)—5. We can conduct the same procedure for the combined evaluation parameters composed from more than two individual parameters. 

After establishing the influence of individual parameters or groups of evaluation parameters on individual diseases, we proceed to estimate the influence of individual evaluation parameters or groups of such parameters on several diseases at the same time (multiple diseases or multimorbidity), using rank statistics [18]. That is to say, after establishing the correlation values of normalized mutual information between individual or combined evaluation parameters with each individual disease, we rank those correlation values and calculate the sum of the ranks for each parameter or group of parameters with all the diseases or medical conditions under consideration. According to the sum of the ranks, we estimate the influence of individual or combined evaluation parameters on multiple diseases at the same time.

### 2.2. Case Materials

To establish and exemplify the proposed method for the evaluation of multiple diseases, in this work, a database of 196 hip fracture patients was analyzed. The patients were treated at the “Shmuel Harofe” Geriatric Medical Center in Beer Yaakov, Israel. Access to the patients’ database was granted in accordance to the principles of the Declaration of Helsinki. The study was approved by the Institutional Review Board of the “Shmuel Harofe” Geriatric Medical Center (IRB approval 52). The patients data used in this study were anonymized before their use. The patients were aged 63 to 97. Seventy-six patients were males, and 120 females. A group of evaluation parameters was selected, out of all the available data on the patients, in order to establish the present new methodological approach. Representative sets of different types of clinical analyses were chosen, focusing on common laboratory biomarkers, including: Cellular and immunological parameters (Lym—number of lymphocytes and WBC—white blood cells), microelements (K—potassium and Na—sodium), hematological measurements (Thr—number of thrombocytes (platelets) and Hg—hemoglobin), metabolic measurements (Gluc—blood glucose) and physiological measurements (pulse—heart rate). The data were collected at admission (ad) and discharge (dis) from the geriatric medical center. Two more discriminating demographic parameters were the patients’ gender and age.

Moreover, according to the patients’ data at admission and discharge, we evaluated the dynamic change and stability of several diagnostic parameters. The dynamic changes were determined as % positive or negative alterations above or below a certain predetermined threshold: Lym + 10%, Lym − 10%, WBC + 1%, WBC − 1%, Na + 1%, Na − 1%, K + 5%, K − 5%, Thr + 10%, Thr − 10%, Hb + 15%, Hb − 15%, Gluc + 10%, Gluc − 10%, Pulse + 5% and Pulse − 5%. The changes within the range of the threshold boundaries might point to the parameters’ stability, while excessive changes may potentially indicate impaired homeostatic/regulatory capability. Thus, the parameter “Lym + 10%” was assigned the value 1, if during the hospitalization, the number of lymphocytes rose by 10% and more, and 0 otherwise. The parameter “Lym − 10%” was assigned the value 1, if during the hospitalization, the number of lymphocytes declined by 10% and more, and 0 otherwise. The thresholds were determined for the whole patients’ dataset, using our earlier reported algorithm, which calculates physiological boundaries by maximizing the value of normalized mutual information [14].

Notably, the present selected parameters are demonstrative, serving to establish the present methodology. Utilizing this methodology, any other parameters and samples can be added in the future for specific analyses and diagnostic models.

Using normalized mutual information, all the selected routine evaluation parameters were correlated with the presence of three common individual age-related degenerative diseases: Dementia, chronic obstructive pulmonary disease (COPD) and ischemic heart disease (IHD). In the present sample, the frequencies of the three diseases, and the frequencies of the combinations of the diseases, were as follows: IHD—0.56; COPD—0.17; DEMENTIA—0.26; IHD+COPD—0.10; IND+DEMENTIA—0.15; COPD+DEMENTIA—0.04 and IND+COPD+DEMENTIA—0.02.

After establishing the informative values of the evaluation parameters and parameter combinations about each individual disease separately, the ranks of the informative values for individual diseases were combined to establish the parameters and parameter combinations that were most informative about the three diseases under examination at once (see the sections “Results” and “Discussion”).

## 3. Results

The proposed method enabled the selection of such evaluation parameters that in combination with each other contain more information about the set of diseases, compared to individual parameters or other parameter combinations. According to the present data, out of the selected individual parameters, it was impossible to select an individual parameter containing significantly more information than another parameter about the three diseases under consideration at the same time. Thus, the best correlation of an individual parameter with an individual disease was achieved for the parameter age in correlation with dementia (the normalized mutual information between those two parameters, NMI = 0.02374), glucose at admission and discharge in correlation with ischemic heart disease (respectively NMI = 0.106 and 0.066), negative dynamic glucose change (Gluc − 10%) in correlation with COPD (NMI = 0.01362) and gender in correlation with COPD (NMI = 0.01362). However, none of these and all the other individual parameters showed a statistically significant correlation with all the three diseases under consideration at the same time (as shown by the Friedman statistical test, see below).

Therefore, to demonstrate the present methodology for the evaluation of multiple diseases, we selected such parameters that, even though individually uninformative, in pairs were correlated in a statistically significant way with all the three diseases under consideration. The nine individual parameters were: The dynamic changes of lymphocytes (indicative of the immune system): Lym + 10%, Lym − 10%, the dynamic changes of thrombocytes (indicative of blood clotting ability): Thr + 10%, Thr − 10%, Na (Na + 1%, Na − 1%), hemoglobin (indicative of blood oxygenation): Hb + 15%, age and gender. From pairs of these individual parameters, 33 combined double parameters were obtained. The data under consideration, representing hospitalized geriatric patients, were rather homogenous. This may be one of the potential reasons why no statistically significant correlations were found between individual parameters and the three diseases. However, out of the 33 double combinations of parameters, it was possible to select the combinations of parameters that statistically significantly contained more information about the diseases under consideration than the other combinations of parameters.

Table 1 presents the values of normalized mutual information (NMI) for the correlation between each of the nine individual parameters with each of the three diseases. As it can be seen, the individual values of correlation were very low. We ranked the values of each NMI entry. The ranks are shown next to the corresponding NMI values in Table 1. The highest rank was nine, where the strongest correlation between an individual parameter and an individual disease was shown, on this specific sample, for the correlation between gender and ischemic heart disease (NMI = 0.02875), and between age and dementia (NMI = 0.02374), and the lowest rank was one for the correlation of lymphocytes change and dementia (NMI = 0.00031) and Na change and IHD and COPD (respectively NMI = 0.00005 and 0.00021).

We now calculated the sum of the ranks for each parameter for all the three diseases under consideration. Hence, we evaluated the effect of single parameters or parameter groups as the sum of corresponding ranks of the NMI. We consider the matrix of NMI ranks as the Friedman statistical model [18], and evaluated the row effect. The Friedman test demonstrated no row effect. This means that, among the individual biomarkers (parameters) under consideration, there was not a single individual marker that would contain more information on the three diseases as compared to other markers. This can be intuitively clarified by the observation that the difference between the highest sum of ranks (21 for gender) and the lowest sum of ranks (11 for hemoglobin, negative lymphocyte change and negative Na change) was rather small. This indicates that the sample was “homogenous”, that is to say, there was little difference in the informative capacity of individual parameters compared to each other. Hence, individual parameters are not suitable as diagnostic markers of the three diseases at the same time. Hence we must proceed to the analysis of combined markers, consisting of two individual markers.

Table 2 shows the values of normalized mutual information between each combined double marker and each disease. Notice the generally higher informative values of double parameters as compared to individual parameters. For example, as an individual parameter, age correlated with heart disease with the NMI value of 0.00018, and gender correlated with heart disease with the NMI = 0.02875, yet together age and gender correlated with heart disease with the NMI equal to 0.02951, which was more than the simple arithmetic sum of the NMI’s (0.02893). This shows a cumulative effect, or improved informative value of age together with gender for ischemic heart disease. It is largely due to such an improved informative value of combined double parameters that we were able to find double parameters that, in a statistically significant way, contained more information about the three diseases than other parameters. This illustrates the unique capability of the information-theoretical methodology to evaluate cumulative or synergistic effects of multiple parameters (diagnostic markers or risk factors) [9,15,16,17].

To prove that we indeed obtained such significantly more informative double parameters, we ranked the NMI entries of Table 2, assigning the best NMI correlations with the largest ranks, presented next to the corresponding NMI values (e.g., rank 33 for the combination of Na + 1% and age for COPD). For each double parameters’ combination, we calculated the sums of the ranks for all three diseases. We then considered the matrix of NMI ranks as the Friedman statistical model, and evaluated the row effect. The Friedman test shows that there was indeed a row effect. This signifies that there was a statistically significant difference between the rows examined. This demonstrates that among the double evaluation parameters under consideration, there were indeed double parameters that contained more information about the three diseases as compared to other evaluation parameters.

Now we compared the different double markers with each other, and create their clustering according to the informative values of those double markers. For the multiple comparisons, the Newman–Keuls test was utilized [19]. We calculated |Rj − Rj+1| > 3.395, where Rj and Rj+1 are elements of the column “Sum of ranks” in the j-th and (j + 1)-th rows of the NMI ranks matrix presented in Table 2, respectively. Using the multiple comparisons method, we established the clustering presented in Table 3. This clustering had several properties: 1) For two neighboring sets presented in Table 3, the smallest element of one set and the largest element of another set positioned nearby were significantly different (α_T_ = 0.05), and 2) there were no differences between elements belonging to the same set (α_T_ = 0.05). Here α_T_ is the probability at least once to detect differences erroneously. Thus, the clustering presented in Table 3 shows that we were indeed able to select double parameters that were more informative about the three diseases under consideration at once, as compared to other parameters. 

## 4. Discussion

In this paper, we developed a new formal methodology for the simultaneous evaluation of multiple diseases, using multiple diagnostic parameters simultaneously. This methodology enables establishing the most informative evaluation parameters and parameter combinations about the presence of several diseases at the same time, showing the cumulative and synergistic effects of such diagnostic parameter combinations. That is to say, we established the most informative double parameters by the strength of their correlation with all three diseases under consideration at the same time, where the strength of correlation was synergistically increased by adding evaluation parameters. In this way, we could examine multiple disease etiology, utilizing the information-theoretical measure of normalized mutual information. The information-theoretical methodology affords unique capabilities for the evaluation of multiple etiologies of multiple diseases (multimorbidities). Thus, information theory provides the capability to estimate cumulative (holistic or synergistic) effects that can be definitive for the emergence of multiple diseases from multiple pathogenic factors. Moreover, the information-theoretical methodology permits the evaluation of any types of parameters, including both discrete parameters (e.g., gender in the present study) and continuous parameters (e.g., blood elements in the present study) in the same model. Moreover, it allows combining, in the same model, any types of diseases, regardless of their domains, for example cognitive and physical impairments. In addition, the information-theoretical methodology allows the evaluation of non-linear relations between diagnostic parameters and diseases, as most commonly occur in clinical practice. The information-theoretical approach is the mathematically grounded approach that uniquely enables all these capabilities [9,15,16,17,20,21]. 

In particular, the information-theoretical methodology principally differs from common types of multiparametric analysis, such as the methods of an analysis of variance (ANOVA) and polynomic regression that are suitable only for the analysis of continuous parameters. The method proposed in the present study is suitable for the simultaneous analysis of both continuous and discrete parameters. In contrast to the information-theoretical method, discretization and approximation in statistical methods, such as ANOVA and linear regression, involve adding new assumptions and information. Furthermore, the methods of an analysis of variance and linear regression establish linear correlations, and hence do not always provide an adequate estimation of non-linear correlations between parameters, unlike the proposed information-theoretical method that can evaluate non-linear correlations. Unlike the present information-theoretical approach, the common statistical measures (such as an ANOVA) do not provide a precise quantitative value (measurement) of the correlation of parameters, but only a determination for the absence or presence of a correlation. Moreover, in attempting to evaluate non-linear correlations, the replacement of linear functions with non-linear functions (introducing a “formula”), as commonly done in the statistical or heuristic methods, does not necessarily solve the problem of potential non-linearity of correlations. First of all, not all non-linear correlations can be presented as a “formula” and secondly, the presentation of a correlation as a formula does not provide any measurement of the value of correlation. In contrast, the information-theoretical analysis does not assume a priori the correlations’ linearity, data distributions’ normality or parameters’ continuity, and provides the precise value of correlation, i.e., the normalized mutual information value in this study.

The development of such an information-theoretical methodology is particularly important for geriatric settings, as the geriatric patients are commonly characterized by multiple diseases with multiple or polygenic etiologies. Thus, for common geriatric diseases, such as Alzheimer’s disease, even for single markers, such as amyloid beta, that are ostensibly strongly correlated with the disease, clinical modifications of those single markers do not necessarily, or even seldom correlate with clinical outcomes [22,23]. Hence, for such diseases, a stronger emphasis on synergistic multiple or polygenic etiologies is recommended [8]. Even a single disease designation, such as “cancer” in geriatric patients commonly involves a composite disease, comprising several types of cancer and accompanying morbid conditions that need to be evaluated simultaneously with reference to multiple risk factors and interventions [24]. Moreover, in geriatric patients especially, in some cases an improvement of a particular type of clinical outcome (e.g., for proliferative diseases such as cancers) may be associated with the deterioration of another concomitant outcome (e.g., degenerative diseases such as Alzheimer’s) [6,7]. Hence an intervention, even though effective for a particular disease, may have adverse or no effects on the multiple diseases or multi-morbidity. The proposed methodology provides a formal theoretically grounded approach that may enable novel capabilities for studying multiple etiologies and risk factors in combination, while evaluating their synergistic (cumulative or holistic) effects, as well as new directions to study multiple clinical outcomes insofar as those outcomes may interact in complex ways potentially being either synergistic or antagonistic. 

Notably, the present work does not yet provide a practicable diagnostic tool, but a direction for future research. Yet, even now, it provided reasonable indications based on the examined parameters routinely available to physicians. At a later stage of research, based on the obtained values of normalized mutual information on large datasets, utilizing new populations and meta-analyses, it may be possible to create assistive diagnostic decision rules involving multiple evaluation parameters [11,25].

This methodology was established here on a limited cohort of geriatric patients, using routinely available evaluation parameters, such as common blood biomarkers and demographic characteristics. Thus, the main purpose of this study was to suggest a new evaluation (correlation) methodology for geriatric assessment. Its further validation and interpretation may be expanded with the use of additional datasets. Yet, even with the present limited dataset, the results of the method’s application may encourage further investigation.

Within the present cohort, the most informative double parameters, with the highest sum of ranks for the tree diseases under consideration, are presented in Table 3, Cluster 1. This is the combination of the positive change of thrombocytes (Thr + 10%) and gender (the sum of ranks—87). The high informative value of this combination may indicate the importance of the blood clotting mechanism differences in males as opposed to females. This finding may tie in with the earlier studies testing the prevention of multiple degenerative diseases via effects on blood clotting (e.g., by low doses of aspirin), and finding that the effects of such interventions in men are different from women [26]. Notably, the differential evaluation of particular parameter values and patterns would require the performance of information-theoretical analysis for specifically selected samples, for example the selection only for men vs. only for women.

Less informative (but still rather high ranking) parameters are in Cluster 2, including the combinations of a large increase of Na and age (sum of ranks—83), age and gender (sum of ranks—80) and increase in thrombocytes and increase in Na (sum or ranks—79). The high informative value of the combination of the relatively large dynamic changes of Na and thrombocytes may indicate the importance of improper or unstable functioning of the blood clotting system for the emergence of multiple age-related diseases. Recent studies emphasized the role of clotting mechanisms, thrombocytes and their secreted factors, for the emergence of several disease states and multimorbidity [27,28,29]. The present result may draw additional attention to this connection, as a possible subject for further investigation.

The combination of age and gender is also informative. Gender by itself turned out to be the most informative for the emergence of multiple diseases, as an individual parameter. This finding may be related with the consistent differences in life expectancy and frailty between men and women [30]. With the addition of age to gender, the information about the emergence of the several degenerative diseases increases even more, demonstrating a “cumulative effect” (the combined markers are more informative than the simple sum of the parameters). Yet, surprisingly, age alone as an individual parameter was among the least informative parameters for the appearance of the three diseases (among the least ranking parameters). This may seem surprising, as the diseases under consideration (ischemic heart disease, COPD and dementia) are well-recognized age-related diseases.

The little informative value of age by itself in the present dataset may be explained by the fact that the examined group is rather homogenous, comprising geriatric patients (aged 63–97) after hip fracture. This may reflect the difficulty of evaluating biomarkers of aging in frail geriatric patients [25,31]. In the present study, data on young and healthy subjects were not available, hence the study necessarily focused on elderly frail subjects as are most commonly found in the clinical settings. Nonetheless, even though not informative by itself, in combination with other parameters in the present elderly cohort, age improved the informative values of the combined markers. 

In our earlier study, we established the information-theoretical methodology to evaluate the weight of each individual parameter (including gender and age) or their combinations (e.g., gender in addition to age) for the emergence of particular diseases (e.g., heart disease). Based on the present data, it was indicated that individual parameters (e.g., total cholesterol) provided little information about a particular disease, but in combination (with the addition of age and gender) their informative value increased substantially [13]. In the present article we proposed the methodology that enables the precise quantitative evaluation of the weight of each individual parameter (such as gender and age) as well as their combinations on multiple diseases at the same time. Here gender and age were among the particular parameters that could contribute to the evaluation of the risk for multimorbidity. Any other relevant parameters could be added in a similar way.

Thus, even within the present homogenous and “difficult” cohort (reflecting the actual clinical geriatric settings, where patients are multi-morbid and are under multiple drug treatments and stress), the information-theoretical methodology was able to select the most informative combinations of biomarkers.

## 5. Conclusions

In this work, we proposed a new information-theory-based methodological approach to determine the most informative individual and combined diagnostic parameters for the simultaneous assessment or prediction of several diseases at the same time. We evaluated the parameters’ informativeness by measuring the strength of correlation of individual and multiple parameters with individual and multiple diseases, using the information-theoretical measure of normalized mutual information. This formal, theoretically grounded measure uniquely provides the precise quantitative value of the strength of correlation, where the correlation may be non-linear, enabling the inclusion of both discrete and continuous parameters, and showing cumulative or synergistic effects of correlation parameters. Such a formal methodology can be particularly helpful for the evaluation of geriatric patients whose diagnosis and treatment are complicated by the presence of multiple aging-related diseases and can help establish the effectiveness of treatments directed against multiple diseases. This methodology proved its applicability for typical clinical analyses, under common clinical conditions for geriatric patients. With additional data, it may be possible to develop such a methodology into a clinically useful diagnostic evaluation tool.

## Figures and Tables

**Table 1:** Highest coefficients and ranks of normalized mutual information (NMI) for the correlation of individual evaluation parameters with three diseases at the same time: Ischemic heart disease (IHD), chronic obstructive pulmonary disease (COPD) and dementia.

Parameters	IHD NMI	IHD Rank	COPD NMI	COPD Rank	Dementia NMI	Dementia Rank	Sum of Ranks
Lym + 10%	0.00202	5	0.00137	7	0.00137	4	16
Lym − 10%	0.00058	4	0.00099	6	0.00031	1	11
Thr + 10%	0.00824	8	0.00688	8	0.01305	8	24
Thr − 10%	0.00015	2	0.00029	2	0.00192	6	10
Na + 1%	0.00649	7	0.00021	1	0.00867	7	15
Na − 1%	0.00005	1	0.00059	5	0.00176	5	11
Hb + 15%	0.00413	6	0.00033	3	0.00042	2	11
Age	0.00018	3	0.00051	4	0.02374	9	16
Gender	0.02875	9	0.01362	9	0.00085	3	21

**Table 2:** Table of coefficients of normalized mutual information for combined double evaluation parameters and their ranks for the three diseases: IHD, COPD and dementia.

Parameters	Parameters	IHD NMI	IHD Rank	COPD NMI	COPD Rank	Dementia NMI	Dementia Rank	Sum of ranks
Lym + 10%	Thr+10%	0.01127	15	0.01697	25	0.01907	20	60
Lym + 10%	Thr–10%	0.00998	14	0.00205	6	0.00702	7	27
Lym + 10%	Na+1%	0.00949	13	0.00518	13	0.01046	12	38
Lym + 10%	Na–1%	0.00371	5	0.00182	4	0.00267	2	11
Lym + 10%	Hb+15%	0.00727	10	0.00197	5	0.00621	6	21
Lym + 10%	Age	0.00271	3	0.00382	9	0.03469	29	41
Lym + 10%	Gender	0.0325	29	0.01695	24	0.01083	13	66
Lym − 10%	Thr+10%	0.01175	16	0.00822	17	0.01399	14	47
Lym − 10%	Thr–10%	0.00082	2	0.00542	14	0.00362	4	20
Lym − 10%	Na+1%	0.01902	21	0.00385	11	0.00943	9	41
Lym − 10%	Na–1%	0.00434	7	0.00433	12	0.01653	18.5	37.5
Lym − 10%	Hb+15%	0.00534	9	0.00154	2	0.03433	28	39
Lym − 10%	Age	0.00734	11	0.01269	20	0.02471	23	54
Lym − 10%	Gender	0.03591	30	0.01834	27	0.00193	1	58
Thr + 10%	Na+1%	0.01931	23	0.02357	29	0.03026	27	79
Thr + 10%	Na–1%	0.01271	17	0.00827	18	0.01562	16	51
Thr + 10%	Hb+15%	0.01584	20	0.00926	19	0.01622	17	56
Thr + 10%	Age	0.02477	25	0.00786	16	0.0393	31	72
Thr + 10%	Gender	0.04089	32	0.02645	31	0.02585	24	87
Thr − 10%	Na+1%	0.01912	22	0.00082	1	0.01933	21	44
Thr − 10%	Na–1%	0.00392	6	0.00176	3	0.00572	5	14
Thr − 10%	Hb+15%	0.00505	8	0.00383	10	0.01404	15	33
Thr − 10%	Age	0.00284	4	0.00249	7	0.02697	26	37
Thr − 10%	Gender	0.03163	28	0.02367	30	0.00316	3	61
Na + 1%	Hb+15%	0.0201	24	0.00323	8	0.00931	8	40
Na + 1%	Age	0.01358	18	0.02859	33	0.04141	32	83
Na + 1%	Gender	0.05063	33	0.01707	26	0.00955	10	69
Na − 1%	Hb+15%	0.01448	19	0.02793	32	0.0239	22	73
Na − 1%	Age	0.00047	1	0.01566	23	0.02659	25	49
Na − 1%	Gender	0.03608	31	0.01517	22	0.01035	11	64
Hb + 15%	Age	0.00788	12	0.00587	15	0.0348	30	57
Hb + 15%	Gender	0.03143	27	0.01861	28	0.01653	18.5	73.5
Age	Gender	0.02951	26	0.01385	21	0.04728	33	80

**Table 3:** Clustering of the combined evaluation parameters.

No	No. of the Set	Clusters	Markers	Markers	Sum of Ranks
1	1.1	Cluster 1	Thr + 10%	Gender	87
2	2.1	Cluster 2	Na + 1%	Age	83
3	2.2		Age	Gender	80
4	2.3		Thr + 10%	Na + 1%	79
5	3.1	Cluster 3	Hb + 15%	Gender	73.5
6	3.2		Na − 1%	Hb + 15%	73
7	3.3		Thr + 10%	Age	72
8	3.4		Na + 1%	Gender	69
9	3.5		Lym + 10%	Gender	66
10	3.6		Na − 1%	Gender	64
11	3.7		Thr − 10%	Gender	61
12	3.8		Lym + 10%	Thr + 10%	60
13	3.9		Lym − 10%	Gender	58
14	3.10		Hb + 15%	Age	57
15	3.11		Thr + 10%	Hb + 15%	56
16	3.12		Lym − 10%	Age	54
17	3.13		Thr + 10%	Na − 1%	51
18	3.14		Na − 1%	Age	49
19	3.15		Lym − 10%	Thr + 10%	47
20	3.16		Thr − 10%	Na + 1%	44
21	3.17		Lym + 10%	Age	41
22	3.18		Lym − 10%	Na + 1%	41
23	3.19		Na + 1%	Hb + 15%	40
24	3.20		Lym − 10%	Hb + 15%	39
25	3.21		Lym + 10%	Na + 1%	38
26	3.22		Lym − 10%	Na − 1%	37.5
27	3.23		Thr − 10%	Age	37
28	4.2	Cluster 4	Thr − 10%	Hb + 15%	33
29	5.1	Cluster 5	Lym + 10%	Thr − 10%	27
30	6.1	Cluster 6	Lym + 10%	Hb + 15%	21
31	6.2		Lym − 10%	Thr − 10%	20
32	7.1	Cluster 7	Thr − 10%	Na − 1%	14
33	7.2		Lym + 10%	Na − 1%	11
